# LiDARPheno – A Low-Cost LiDAR-Based 3D Scanning System for Leaf Morphological Trait Extraction

**DOI:** 10.3389/fpls.2019.00147

**Published:** 2019-02-13

**Authors:** Karim Panjvani, Anh V. Dinh, Khan A. Wahid

**Affiliations:** Department of Electrical and Computer Engineering, University of Saskatchewan, Saskatoon, SK, Canada

**Keywords:** phenotyping, LiDAR, leaf traits, leaf area, leaf length and width, low-cost phenotyping, 3D phenotyping

## Abstract

The ever-growing world population brings the challenge for food security in the current world. The gene modification tools have opened a new era for fast-paced research on new crop identification and development. However, the bottleneck in the plant phenotyping technology restricts the alignment in geno–pheno development as phenotyping is the key for the identification of potential crop for improved yield and resistance to the changing environment. Various attempts to making the plant phenotyping a “high-throughput” have been made while utilizing the existing sensors and technology. However, the demand for ‘good’ phenotypic information for linkage to the genome in understanding the gene-environment interactions is still a bottleneck in the plant phenotyping technologies. Moreover, the available technologies and instruments are inaccessible, expensive, and sometimes bulky. This work attempts to address some of the critical problems, such as exploration and development of a low-cost LiDAR-based platform for phenotyping the plants in-lab and in-field. A low-cost LiDAR-based system design, LiDARPheno, is introduced in this work to assess the feasibility of the inexpensive LiDAR sensor in the leaf trait (length, width, and area) extraction. A detailed design of the LiDARPheno, based on low-cost and off-the-shelf components and modules, is presented. Moreover, the design of the firmware to control the hardware setup of the system and the user-level python-based script for data acquisition is proposed. The software part of the system utilizes the publicly available libraries and Application Programming Interfaces (APIs), making it easy to implement the system by a non-technical user. The LiDAR data analysis methods are presented, and algorithms for processing the data and extracting the leaf traits are developed. The processing includes conversion, cleaning/filtering, segmentation and trait extraction from the LiDAR data. Experiments on indoor plants and canola plants were performed for the development and validation of the methods for estimation of the leaf traits. The results of the LiDARPheno based trait extraction are compared with the SICK LMS400 (a commercial 2D LiDAR) to assess the performance of the developed system.

## Introduction

An estimated 30% rise in number of people on the planet by 2050 will bring new challenges, one of those challenges is the need of enough food, which is estimated to increase about 1.5 times of that of today (Yuan et al., 2004; Tilman et al., 2011). To meet this goal, it is required to increase the yield of the crops that are produced. There are several ways to improve the yield of crops, specifically advances in gene modification and mutation have provided huge opportunities for the improvements in the quality and quantity of production capability of the crops (Uzogara, 2000). Understanding the interaction of genotype with environment is of prime importance, which can be achieved by the measurement of phenotypic traits of the crop (Houle et al., 2010; Großkinsky et al., 2015). The field of phenomics is large-scale collection of phenotypes to study, analyze and understand the interaction of genomic variations with varying environment by revealing the relation between genotype and phenotypes (Allen et al., 2010; Houle et al., 2010; Nsf-Usda, 2011). Traditionally, plant phenotyping has been achieved by manually collecting the phenotypes from the plants to select the best individual variety (Walter et al., 2015). Technological advancement in the plant phenotyping has been a topic of interest among interdisciplinary researchers in recent years. The efforts have been put into using and optimizing the available technologies to adapt to the need of plant phenotyping (Fiorani and Schurr, 2013). Active sensors such as Greenseeker RT 100 (NTech Industries Inc., Ukiah, CA, United States) and the Crop Circle ACS-470^®^ (Holland Scientific, Inc., Lincoln, NE, United States) have been used in studying the biochemical traits (e.g., nitrogen uptake) of plants (Winterhalter et al., 2013). Moreover, use of imaging sensors have been explored to find the relation between the genome and the environment (Walter et al., 2015). However, most of the developments have been focused on lab experiments with some for in-field experiments, but are unavailable commercially at large scale.

Plant imaging using a 2-dimensional (2D) color – visible light spectrum (VIS) – cameras were used by numerous researcher to develop a plant trait characterization algorithms (Furbank and Tester, 2011; Fahlgren et al., 2015). However, the VIS cameras are prone to the lighting conditions and might perform differently under changing lighting conditions, leaf shadows, overlapping leaves and differentiating leaves from the soil background (Li et al., 2014). A 3-dimensional (3D) reconstruction of the canopy root and shoot architecture can provide detailed view of the plant structure and distribution of organs. Technologies such as tomography and 3-D imaging can be utilized for generating a 3D models for analysis. Tomographic imaging such as Magnetic Resonance Imaging (MRI) and X-ray Computed Tomography (CT) can provide detailed information of the root and shoot architecture and distribution (Stuppy et al., 2003; Van As and van Duynhoven, 2013). However, the tomographic imaging is bulky and is low-throughput (Li et al., 2014). 3D imaging can provide the detailed view of the plant structure above-ground by using technologies such as Time of Flight (ToF), Light Detection and Ranging (LiDAR), and Stereo Vision cameras to create a detailed map of the vegetation and canopy structures (Li et al., 2014). While ToF cameras can be considered ideal for high-throughput 3D data acquisition due to their high frame rate, they are influenced by the sunlight as well as their low resolution limits the adaptation in phenotyping applications. On the other hand, 3D model reconstruction from the multi-perspective 2D images (stereo vision) is possible, but the process of generating 3D model is highly dependent on the quality of the 2D images, which suffers from the illumination conditions. In addition, extensive calibration for the 2D cameras is required to estimate the 3D models. For instance, 3D photogrammetry using 2D images requires many high-resolution images taken from different angles and high level of calibration. Furthermore, the algorithms for processing the images to generate a 3D model are computationally expensive, requiring the use of high-end processors and huge amount of available memory due to sophisticated algorithms for phenotype extraction. On the other hand, LiDAR can provide the phenotypic information accurately with reasonably easy processing steps. However, LiDAR sensors are expensive and bulky. The fact that available technologies are expensive, monetarily and/or computationally, limits the exploration by the research community at large. Moreover, the LiDAR is best known for the 3D model reconstruction of the canopy due to its accuracy, robustness, and resolution. LiDAR uses a its own laser light source to estimate distance to reflecting object. In plant phenotyping, several attempts toward the reconstruction of the canopy have been made. Reconstruction of the 3D model allows for the analysis of the complex traits, such as shape, area, and alignment of the leaves.

LiDAR became known to general public in the early 1970s when the astronauts used it to map the surface on the moon. Since then, LiDAR has been used in remote sensing applications and generally involves data acquisition with an airplane or helicopter while combining the range data with GPS to map the them to geolocation (National Oceanic and Atmospheric Administration, 2014). The LiDAR’s accurate distance estimation and ability to map the structure makes plant phenotyping community believe that LiDAR can provide an opportunity to look at the plant with more accurate 3D modeling, revealing the critical geometric parameters of the plants. The most frequently used 2D LiDAR collects two-dimensional information, generally using a rotating mirror, at a very high speed. The two notable developments in LiDAR-based hardware systems are the PlantScan plant phenotyping systems developed by Australian Plant Phenotyping Facility and PlantEye (costs about Canadian $50,000 for scanner) phenotyping system by PhenoSpex. Other developments on LiDAR-based technology are mostly utilization of LiDAR data for developing methods to extract plant traits (Lin, 2015). Various attempts to utilizing the commercial LiDAR sensors such as LM400 (Sick Inc.) have been made in study of developing new techniques for 3D reconstruction of the canopy. Moreover, 3D data analysis algorithms are generally modified to meet the requirement of the trait extraction. The 3D model of the canopy can be constructed while moving the 2D LiDAR along the direction of the scanning plane. While 3D model constructed using LiDAR scans is not as dense as those constructed using 2D images, the scans can provide useful information for extracting the plant morphological traits (Rosell et al., 2009; Bietresato et al., 2016; Sun et al., 2017). Deery et al. (2014) have included the 2D LiDAR (LMS400, Sick AG, Waldkirch, Germany) in their field-based phenotyping platform, Phenomobile, for estimating the canopy height in the different height genotypes of the wheat with high correlation with manually measured height concluding that may be LiDAR is potential best alternative to VIS imaging. In a similar study, Tilly et al. (2012), used the terrestrial laser scanner to measure the height of the rice crop with highly accurate estimates. Rosell et al., (2009) have validated the feasibility of 2D terrestrial LiDAR to reconstruct the 3D model of the canopy, concluding that it can reveal essential structural and geometric traits of the plants. In (Sun et al., 2017), the authors use the 2D LiDAR to construct the 3D point cloud and achieving more than 90% accurate height estimates. Omasa et al. (2006), in their study of 3D LiDAR imaging for understanding plant responses, concludes the potential application of LiDAR in understanding of plant’s response to stress. In (Hosoi and Omasa, 2009), authors have obtained the high correlation between LiDAR estimated leaf area and dry weight of the leaf in the wheat. Recently, in the past couple of years, the feasibility of LiDAR to construct the 3D model and analyze the 3D to understand structural variety in the plants has drawn attention from many researchers dealing in the plant phenotyping field. In one of the recent studies, (Jimenez-Berni et al., 2018), authors integrated the LiDAR on the high-throughput phenotyping platform, Phenomobile, to non-destructively estimate the wheat characteristics such as height, ground cover, and above-ground biomass by comparing it with RGB and NDVI data. Herrero-Huerta et al., (2018) have monitored the leaf movement activity in the indoor plant using terrestrial LiDAR, revealing the angles of leaf movement under various lightning conditions. Following the established methods in Sun et al. (2017), the authors performed the in-field experiments for growth analysis for cotton plants in Sun et al. (2018). The analysis of the plant morphological traits such as height, projected canopy area, and plant volume were extracted from the LiDAR data. The above ground structure of a plant is an essential characteristic to evaluate the plant’s ability to resist environmental changes and diseases. Moreover, the above ground organism of the plant is responsible for the process of the photosynthesis – apparently, one of the most critical traits to estimate the yield – and growing the fruit or seeds. Leaf area, leaf expansion, and the ground cover are some of the traits that can be used to estimate the photosynthetic rate (Reich et al., 1998).

Most of the available technologies and platforms are still in the research phase and are not ready for commercial use, and those available commercially (such as PlantEye by PhenoSpex B.V., Netherlands) are highly expensive, inaccessible and bulky. Hence, there is a need to develop the cost-effective solutions for the phenotyping. This work addresses the main challenge of developing a cost-effective LiDAR-based 3D scanning system for the estimation of the key leaf traits (length, width, and area).

## Materials and Methods

### Plant Material

The laboratory experiments were performed on different plants. In the first experiment, five different indoor plants from three different families were used. In the second experiment, three plants of canola were used as scan subjects.

**FIGURE 1:**
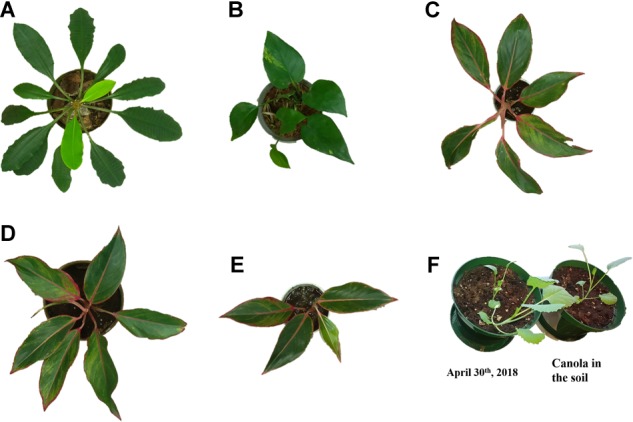
Digital images of the indoor plants used for the experiment in the laboratory: **(A)** an arbitrary wild plant, **(B)** orchid, **(C–E)** Aglaonema plants, and **(F)** Canola grown in laboratory.

In the first experiment, plant varieties include Orchid, Aglaonema and an arbitrary wild plant, which are readily available from gardening stores. Total of five plants have been brought to a laboratory and was given adequate water every 2 days. There were three different plants of Aglaonema with varying sizes and leaf numbers. Figure 1A–E shows a digital image of all five of them. All three different species of plants have varying leaf shape and sizes. The images shown in the figure are taken from the top of the plants using the raspberry pi camera module.

For the second experiments, canola plants were used. The canola seeds were soaked in regular drinking water for 2 days and the seed were transferred to a pot. Approximately a week after transferring to the pot, canola started emerging. Pictures of the canola on April 30th, 2018 is shown in Figure 1F. The experiment was performed on the canola plants for 3 weeks. Canola has more compound leaves and is hard to phenotype due to surface curvature and non-uniform structure of leaves. The indoor plants are used for the development and canola plants for validation of the post-processing algorithms and software.

### Low-Cost LiDAR Based Scanning System

**FIGURE 2:**
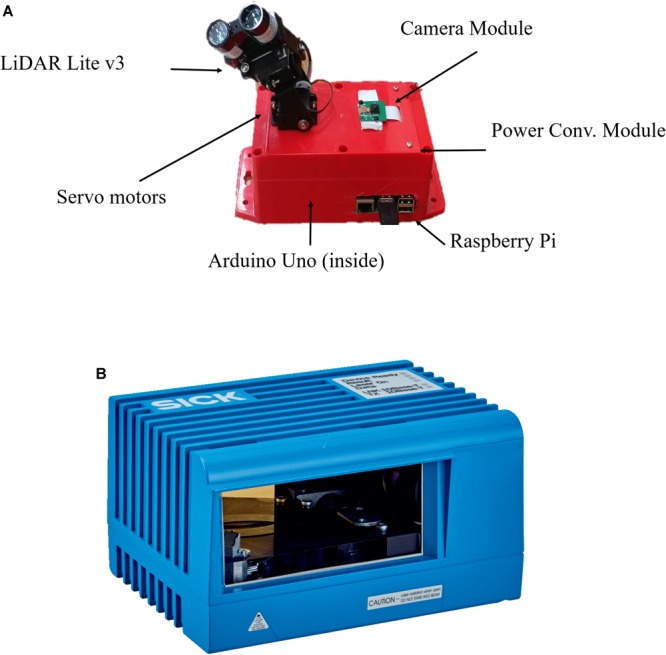
**(A)** a low-cost LiDAR based design that includes Arduino Uno, Raspberry Pi, servo motor-based mechanism and a low-cost LiDAR sensor. **(B)** A commercial 2D LiDAR scanning system LMS400-2000 (Sick Inc., United States).

A low-cost Light Detection and Ranging (LiDAR) sensor LiDAR-Lite v3 (Garmin Ltd., United States) has been utilized to build a system. Moreover, this low-cost distance measurement sensor has been interfaced with Arduino Uno and Raspberry Pi (model 3B) to utilize already developed programming libraries, which ensures fewest coding and easy operation. The low-cost LiDAR based scanning system (LiDARPheno) is shown in the Figure 2A. The system works as a scanner setup with control over horizontal and vertical field-of-view (FoV) and stepping angle between two point acquisitions.

LiDAR works on the principle of Time of Flight (ToF) and uses time of transmission and reception of a laser light to calculate distance from device to reflecting object. Distance is measured by multiplying speed of light with half of time-of-flight. LiDAR-Lite v3 has a detection range up to 40 m and can measure distances with accuracy of ±2.5 cm. This low-cost LiDAR sensor is a low power device with current consumption of up to 130 mA and operates with laser light wavelength of 905 nm.

Arduino Uno is an open hardware platform which is popular for prototyping of small systems. The Arduino controller is used to control the scanning operation. Moreover, manufacturer of LiDAR-Lite v3 provides Arduino library for single distance measurement. Due to low power requirement and ease of use, it is ideal for development of the low-cost 3D plant phenotyping system. Raspberry Pi is a mini-computer operating on Linux distribution (Raspbian OS). In this system, the minicomputer (Raspberry Pi 3 Model B) works as main control element of all the operations. For the system to be more flexible and eliminate, a desktop system is used as a main controlling device. It has capability to connect to the internet which makes this low-cost system wireless. The hardware of the system is designed in such a way that it can provide control over the field-of-view of the scan. This means, user has full control over how much of the scanning is required for a particular scene to capture. User can configure the horizontal and vertical FoV for the scan (up to 180 degree).

Two micro servo motors (HiTec HS85BB) provides control over horizontal and vertical scanning patterns. Micro servo brackets are used to hold the servo motors and the LiDAR sensor on top of that. All of the system components are housed in one box, making it a wireless and ready-to-use instrument for scanning.

A Raspberry Pi camera module is used to take a reference RGB image of the scene before the scan is initiated. Reference image provides the detail about the scene, which in turn can be used to verify that the scan was successful. A power module is used to convert any Direct Current (DC) input voltage from 5 to 12 V DC to 5V DC, making it usable with any power adaptor or battery. In this system, a 7.4 V, 2000 mAh, 5C Lithium Polymer battery is used to power the system. Experiment results show that the battery lasts up to 2 h with continuous operations.

The primary reason behind using the raspberry pi and Arduino together, even though raspberry pi is capable of doing the job, is to utilize the already developed and publicly available libraries. For instance, manufacturers of the LiDAR device provides the library to interface it with Arduino and APIs can be easily installed on the raspberry pi to get the functionality of uploading data. Moreover, as part of the future development plans, authors would like to utilize raspberry pi as the single point processing unit, which can process data as it is acquired and finally upload the results to the desired server. On top of that, if necessary, an independent scanning system with Arduino alone could be attached to central raspberry pi to make a network of the systems. This arrangement provides low code development time, plug-and-play operation, simple processing algorithms and cost-effective arrangement.

### Commercial LMS400-2000 LiDAR

Commercial LiDAR, LMS400-2000 (Sick Inc., United States), has been used to assess the performance of the low-cost design. LMS400-2000 operates on 650–670 nanometer (nm) visible laser light to estimate distance to reflecting object using the principle of ToF. This LiDAR has been used to track and assess quality of the product in production lines. Moreover, authors in (Jimenez-Berni et al., 2018) have used this LiDAR for estimating plant height, ground cover and similar plot level traits from the field. A photo of this commercial LiDAR system is shown in Figure 2B.

### Data Acquisition

#### Acquisition Setup for Experiments

**FIGURE 3:**
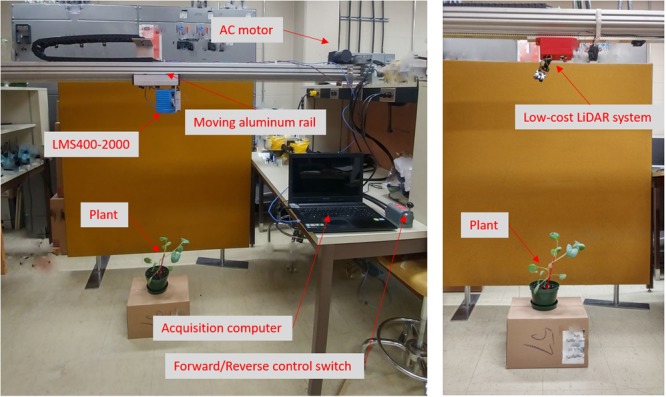
LiDAR Data Acquisition setup in laboratory. **(Left)** Setup with commercial LMS400-2000 LiDAR. **(Right)** Setup with low-cost LiDAR system (LiDARPheno).

The acquisition setup in the laboratory environment is shown in Figure 3. The conveyer belt based mechanical setup for LMS400-2000 consists of:

(1) An aluminum railing which can support weight of LiDAR system combined with conveyer belt and an alternate current (AC) motor with forward and reverse switched control has been built by our collaborator from mechanical engineering. It provides control over forward and reverse motion of the attached device for scanning and data acquisition purpose. The setup moves at constant speed of 18.0724 cm s^-1^.
(2) The mount for LiDAR is made from aluminum sheet capable of holding LMS400-2000 and providing access to power and Ethernet cable. This makes LiDAR data accessible from a computer that can be placed far away.
(3) Commercial LiDAR has been attached to this setup when data acquisition was performed. At a scanning frequency of 360 Hz (scans per second), each scan is 0.5 mm apart.

For data acquisition with LiDARPheno, system itself has horizontal and vertical movement control, which eliminates need for the moving setup. For a simplicity, our device is attached to the aluminum railing with Velcro (hook and loop fastener).

#### Data Capture With LMS400-2000

A library containing functions for data acquisition from LMS400-2000 has been developed by our collaborators based on the publically available library from Robot Operating System (ROS^1^). The programming language Python 2.7 (Python Software Foundation^2^), an easy to use and learn scripting language, has been used to develop this library. This library is used to write a data acquisition script using python. The data containing reflectance and distance information is stored in a two different Comma Separated Values (CSV) file concurrently. LMS400-2000 provides access to distance information as well as reflectance information (i.e., reflectivity of a reflecting target surface). Reflectance information along with distance values are largely useful in separation of plant material from other objects. Jimenez-Berni et al., (2018) have shown that reflectance values higher than 5 are generally non-plant objects and can be used to separate plant points from non-plant points.

#### Data Capture With LiDARPheno Scanning System

**FIGURE 4:**
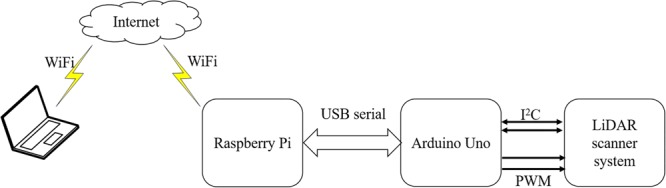
A data flow diagram of data collection using low-cost LiDAR design.

This low-cost LiDAR based design is truly wireless and can be remotely operated by taking advantage of the wireless connectivity already integrated in the Raspberry Pi mini-computer. The system connects to available Wi-Fi and user can control it via remotely located computer system. Once the user command is received, the system starts scanning the scene. The data being captured are LiDAR distance data, reflectance information and digital image of the scene. Once acquired, it automatically sends all the data to predefined Dropbox^3^ (Dropbox, Inc.) folder. Distance and reflectance information is stored in CSV file whose name is according to the time and date of the scan. The acquired digital image is also uploaded along with CSV files so that data from distance can be compared to the digital image. Figure 4 shows the flow diagram of the data acquisition using low-cost design.

A user initiates a command via remote terminal (PC or smartphone) to scan the scene, raspberry pi creates files for storing data and forward the command to Arduino via Universal Serial Bus (USB) communication and then Arduino communicates to LiDAR scanning using Inter-Integrated Circuit (I2C) communication protocol and controls the servo motors using Pulse Width Modulated (PWM) signals. Once the data are ready, Raspberry Pi uploads all the data to a remote file storage server in DropBox.

#### Ground Truth Data Acquisition

**FIGURE 5:**
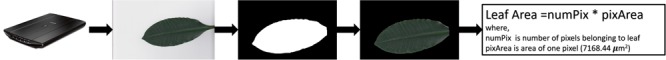
Ground truth leaf area acquisition procedure. From **(left)** to **(right)**: Leaf is scanned using a document scanner Canon LiDE 220, leaf is then separated from background in scanned image using color threshold, number of pixels belonging to leaf are counted and then multiplied with area of each pixel to obtain the leaf area.

For this study, the length, width and area of an individual leafs of the plant are estimated. Hence, the ground truth is also acquired at the time of scan so that it can be compared with the estimated values in the later stage. Leaf length and width are manually acquired with measure tape, while leaf area is calculated by scanning each leaf with Canon LiDE 220 (Canon^4^) document scanner. A document scanner flattens the leaf while scanning it, which ensures the whole leaf area has been exposed to the scanner. Leaves of each plant are scanned at resolution of 300 dots per inch (dpi), which if calculated accounts to ∼7168.44 μm^2^/pixel. All the pixels belonging to leaf (i.e., leaf color pixels) are added and multiplied with the area of each pixel. This method is also confirmed using a centimeter graph paper to estimate the area of a square, which gives about 99.98% accurate area calculation. A sample of one of the leaf scans and procedure is shown in Figure 5.

### Data Analysis/Processing

A CSV file is imported into MATLAB R2017a^®^ environment (MathWorks, United States^5^). CSV file contains polar distance from the sensor to the reflecting surface and hence needs conversion to Cartesian coordinate system.

#### Conversion to Cartesian Coordinate System

As the low-cost LiDAR system is steady system and horizontal and vertical angles are known from the user specified FoV, range data acquired with low-cost LiDAR based system are converted using following Equations 1, 2, and 3.

(1) $$\begin{array}{c} X=rho^{*}\cos(\phi ) \end{array}$$

(2) $$\begin{array}{c} Y=rho^{*}\sin{(\phi )}^{*}\cos(\phi ) \end{array}$$

(3) $$\begin{array}{c} Z=rho^{*}\sin{(\phi )}^{*}\sin(\phi ) \end{array}$$

where:

• “rho” is polar distance between reflecting surface and a sensor.
• ϕ is Azimuth (vertical) angle of scan for particular point.
• 𝜃 is Elevation (horizontal) angle of scan for particular point.

On the other hand, LMS400-2000 has only one rotating mechanism that is horizontal movement angles and hence does not require the full conversion. In the experiments, *X* is assumed to be the values of the moving part, i.e., start of scan is 0 cm and each line scan is 0.5 millimeters (mm) apart. Hence, only *Y*- and *Z*-values needs to be converted from the polar distance. This conversion is performed using Equations 4 and 5.

(4) $$\begin{array}{c} Y=rho^{*}\cos(\phi ) \end{array}$$

(5) $$\begin{array}{c} Z=rho^{*}\sin(\phi ) \end{array}$$

**FIGURE 6:**
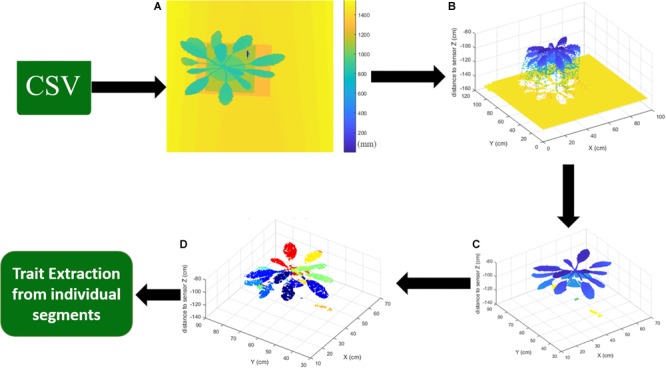
A sample of post-processing of acquired data. **(A)** Raw distance data from CSV file represented as an image. **(B)** Range data converted to Cartesian coordinate system. **(C)** After refine/filtering the noise and outliers along with distance threshold to remove the background scene. **(D)** Segmented point cloud to identify each individual leaf.

Once converted to Cartesian coordinate system, *X, Y*, and *Z* represents corresponding coordinates in real-world system in centimeters (cm). These coordinates can be plotted using 3D scatter plot to visualize a point cloud of the scene. Figure 6A,B shows a raw data represented as a false color image and sample 3D point cloud of one of the scanned indoor plants, respectively.

#### Point Cloud Cleaning/Filtering and Outlier Removal

LiDAR range data tends to be noisy and have outliers because of the reflectance near the edges, reflectance property of an object, inclination angle of an object surface, and environmental parameters (such as light intensity). Hence, it is required that the acquired point cloud be processed and filtered through a filtering algorithm. Before the data cleaning/filtering is performed, the background is removed from the point cloud. To reduce the computation for the filtering algorithm, a height and reflectance value-based threshold has been applied to remove the pints belonging to the background. For data captured with LMS400-2000, a red reflectance has been used to threshold and remove non-plant objects from the scan. As discovered by Jimenez-Berni et al. (2018), reflectance from any vegetation is about 5 Digital value. However, in the experiments, number 20 was found to be better threshold for removing non-plant objects from the scan. This step of applying range/reflectance threshold ensures that the background points are not processed, and the data processing remains simple rather than complex algorithms. At this step, point cloud contains data captured from plant surface and can be used to segment (differentiate) each leaf after the data filtering operation has been performed.

In the experiments, the point cloud data were processed using various filtering algorithms including bilateral filter (Digne and de Franchis, 2017), but due to the nature of the plant leaf’s surface, they failed to perform satisfactorily. Various outlier removal and filtering algorithms have been studied and adapted for Airborne LiDAR data such as multi-directional (Meng et al., 2009b), multi-resolution (Silván-Cárdenas and Wang, 2006) and morphology-based (Meng et al., 2009a) algorithms for building detection and digital terrain map (DTM) generation (Breunig et al., 2000; Chen et al., 2007, 2012, 2017; Kobler et al., 2007). However, the airborne LiDAR data is generally in meters and centimeter-level resolution is hard to find in any airborne LiDAR data. Hence, a neighborhood-based filtering method should be developed that can be easy to implement and process.

Neighborhood based filtering has been used widely in the field of image processing, but authors were unable to find one that can work satisfactory due to the noise levels and nature of leaf surface curvature. Moreover, plant leaves tend to absorb most incident light in the 600 nm range (same as LMS400), which could be a major factor in noise levels. The reason for choosing neighborhood-based method for processing the LiDAR data is that there is more likelihood of points’ relation to each other in neighborhood. Also, the ability to differentiate the outlier point in the region becomes relatively simple, as outliers tend to have more difference to neighboring points, according to our data analysis. The main idea behind this filtering algorithms is to find the neighbor points based on user-defined window side, like a mean filtering algorithm in digital image processing. For each point in the point cloud, algorithm finds its neighborhood based on the user-defined 3D window and number of points within that 3D box. If neighbor points within that window are more than the user-defined threshold, then the point is refined based on the average height of the neighboring points otherwise that point is discarded. Hence, it provides functionality of both, point cloud filtering and outlier removal, in one simple algorithm. In the experiments, two iterations of this algorithm were used to refine the obtained point cloud. Parameters (3D voxel size and number of neighbors) can change for different leaf structures and sizes. Result of data point cleaning and filtering is shown in Figure 6C.

#### Point Cloud Segmentation for Individual Leaves

The segmentation is process of identifying each individual object from the image/point cloud. The modified region-growing algorithm was used for segmentation of each leaf. Region-growing is a simple neighborhood-based algorithm that determines whether neighbors belong to the region or not. Conventional region growing segmentation algorithm (for image processing) requires a seed (pixel) to be selected beforehand and then the algorithm segments the image in different regions. In our modified algorithm, it not only selects seeds itself (the first point is selected as seed automatically), but it also works with 3D point clouds. However, the algorithm segments the data is a slow process and hence it must be improved. It was improved to work with the so-called OcTree data structure. This increases the processing time by a huge amount because of the fact that instead of processing all the data points in the point cloud, a block of points is processed. This process of individual leaf segmentation results in each leaf identified and provides set of points belonging to a particular leaf.

### Trait Extraction

Segmentation process identifies each leaf and different color labels are assigned to each leaf as can be seen from Figure 6D. These individual segments are treated as each leaf and fed to the trait extraction module, where different traits are estimated using the point cloud data. In this paper, our focus was on extraction of leaf length, leaf width and leaf surface area. Methods of extracting each traits is explained in the following subsections.

#### Leaf Length/Width Extraction

Extraction of leaf length from each of the segment was tricky part as each leaf might have different orientation, size and structure. Curve fitting is used on *X-* and *Y*-coordinates of the segmented points for this purpose. First, the orientation of leaf is estimated using the minimum and maximum values of the *X*-and *Y*-coordinates of the segment. The absolute difference between minimum and maximum values provides the distance between these two. If the leaf is oriented along *X*-axis, distance value between minimum and maximum of *X*-axis will be the highest and vice versa. Then a polynomial of degree 2 is applied to fit *X-* and *Y*-coordinates which results in an Equation (6).

(6) $$\begin{array}{c} Y=a*X^{2}+b*X+c \end{array}$$

where:

• *X* is vector of *X*-coordinates in the segment containing 3D data points.
• *Y* is vector of *Y*-coordinates that can be estimated using the equation.
• *a, b*, and *c* are constants that are obtained using polynomial fit to *X* and *Y* data.

Now if the leaf is oriented along *X*-axis, 50 equally spaced samples are taken between minimum and maximum value of *X*-coordinates in that segment and corresponding *Y*-coordinates are estimated and vice-versa. With these obtained *X*-and *Y*-coordinates, nearby points from the original segment are obtained to get a straight line between minimum and maximum value of the *X-* or *Y*-coordinate. After that Euclidean distance between each point of the obtained line is calculated using Equation (7).

(7) $$\begin{array}{c} Euclidean\_distance\left(a,b\right)=\sqrt{{\left(a_{x}-b_{x}\right)}^{2}+{\left(a_{y}-b_{y}\right)}^{2}+{\left(a_{z}-b_{z}\right)}^{2}} \end{array}$$

where, “*a*” and “*b*” are two points in a 3D space and ax, ay, az and bx, by, bz are corresponding *x-, y-*, and *z*-coordinates of point “a” and for “b”.

**FIGURE 7:**
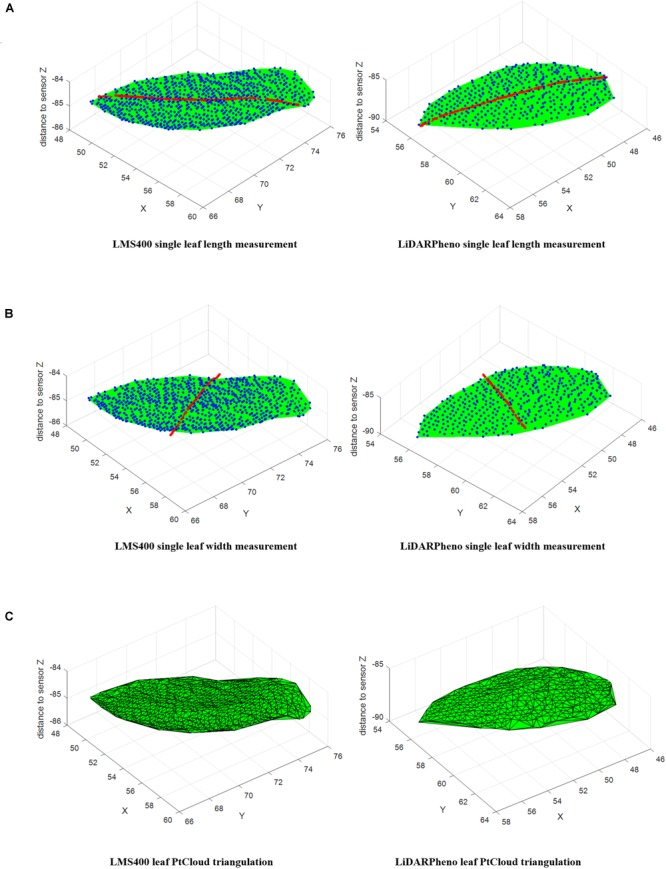
Measurement methods on individual leaf point cloud data: Leaf Length measurement on LMS400 and LiDARPheno data **(A)**. Leaf width measurement on LMS400 and LiDARPheno acquired point clouds **(B)** Delaunay triangulation for leaf area measurement on LMS400 and LiDARPheno point clouds **(C)**.

All these Euclidean distances are added together, which results in the length of the leaf. This process of obtaining leaf length is repeated for all the segments (leaves). Figure 7A presents the leaf length measurement using the above-mentioned method of curve fitting. The samples used for leaf length measurement are shown as red-colored dots in the point cloud. Same method is used for leaf width estimation, the change being plan will change from XY to YX and vice versa. The points obtained for width are shown in Figure 7B.

#### Leaf Area Extraction

Leaf surface area estimation is a different process than estimation of leaf length and leaf width. Data points for each leaf are available, which can be used to estimate the leaf surface area. In this work, a widely accepted Delaunay triangulation (Delaunay, 1934) method is used for generating triangles or surface from 3D point cloud data. The MATLAB function *delaunayTriangulation* is used for generating triangles from the 3D data points^6^. Then area of each triangle is calculated and added to get the area of the surface. For any three points *A*(*x, y, z*), *B*(*x, y, z*), and *C*(*x, y, z*) in a 3D space, surface area of that 3D triangle can be calculated using^7^ Equation (8).

(8) $$\begin{array}{c} Area\left(A,B,C\right)=\frac{1}{2}\;\sqrt{{\left|\begin{array}{ccc} A_{x} & B_{x} & C_{x}\\ A_{y} & B_{y} & C_{y}\\ 1 & 1 & 1 \end{array}\right|}^{2}+{\left|\begin{array}{ccc} A_{y} & B_{y} & C_{y}\\ A_{z} & B_{z} & C_{z}\\ 1 & 1 & 1 \end{array}\right|}^{2}+{\left|\begin{array}{ccc} A_{z} & B_{z} & C_{z}\\ A_{x} & B_{x} & C_{x}\\ 1 & 1 & 1 \end{array}\right|}^{2}} \end{array}$$

Area of each triangle generated using Delaunay triangulation are calculated and added to get the final surface area of the leaf. Figure 7C shows the Delaunay triangulation of the point cloud data to estimate the area of the leaf. As mentioned above, area of the individual triangle is calculated and then all those areas are summed up to finally get the area of the leaf.

#### Accuracy Assessment

**FIGURE 8:**
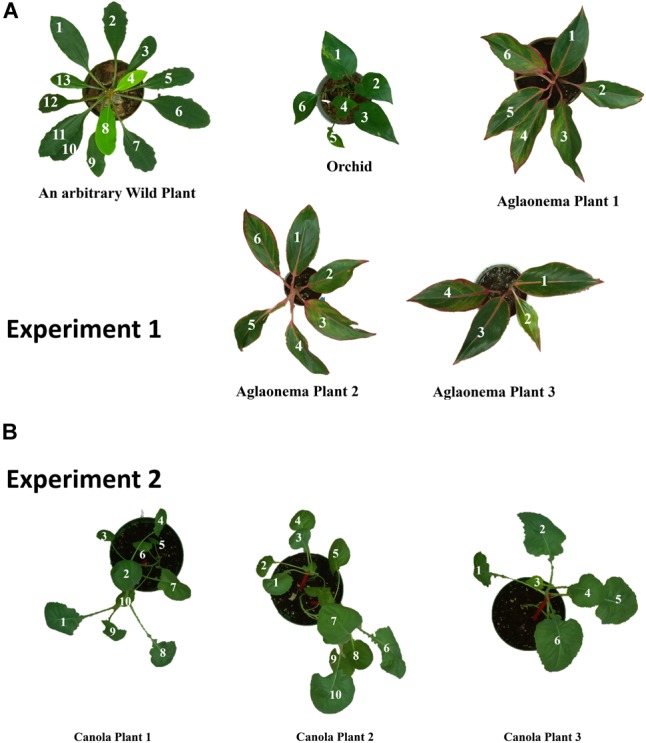
Leaf number annotation for plants in **(A)** experiment 1, and **(B)** experiment 2.

Absolute Percentage Error (APE) is used to evaluate the results of the estimation of the leaf traits (length, width, and area). Two experiments were performed in this work, one is on the indoor plants shown in Figure 8A and another, for validation, on canola plants presented in Figure 8B. Equation (9) is used to calculate the percentage error of estimation.

(9) $$\begin{array}{c} APE=\frac{\left|Actualvalue-Estimatedvalue\right|}{Actualvalue}*100 \end{array}$$

The relation between the ground truth and estimation results can be best represented using the linear correlation plot. In this work, the Root Mean Square Error (RMSE) and coefficient of determination (*r*^2^) are used to represent the relationship between the ground truth data acquired using the manual measurement of the leaf length and the estimated leaf length using the LMS400 data. RMSE was calculated using the Equation (10).

(10) $$\begin{array}{c} RMSE=\sqrt{\frac{\sum_{i=1}^{n}{\left(groundTruth_{i}-estimate_{i}\right)}^{2}}{n}} \end{array}$$

## Results and Discussion

### Leaf Number Annotation

An RGB images taken with raspberry pi camera module are used as reference to assign a number to individual leaf for referencing the estimated traits to the leaf individually. For experiment 1, the annotated leaf numbers are shown in Figure 8A. Generally, leaf numbers are given in the clockwise direction. For example, if the result table refers to the leaf 1 of an arbitrary wild plant, the leaf annotated with number 1 is being referred. Also, the annotated leaf number was used in the auto-calculation of the error rate and generate a report in the form of an excel file. Similarly, Figure 8B shows the leaf number annotation for the experiment 2 of this study.

### Leaf Length Extraction Results

Experiment 1 consisted of five plants with three different species of indoor plants. Table 1 shows the error rates of the leaf length estimation using LMS400 and LiDARPheno.

**Table 1:** Leaf length estimation results.

		Minimum	Mean	Maximum	Segmentation
		% error	% error	% error	rate
**Arbitrary wild plant**	**LMS400**	1.03	8.33	27.83	1.00
	**LiDARPheno**	2.24	11.86	29.70	0.77
**Orchid**	**LMS400**	0.08	22.85	37.54	0.83
	**LiDARPheno**	5.44	20.75	35.03	0.83
**Aglaonema plant 1**	**LMS400**	0.58	9.08	29.30	1.00
	**LiDARPheno**	5.19	18.43	38.45	1.00
**Aglaonema plant 2**	**LMS400**	2.65	10.52	36.10	1.00
	**LiDARPheno**	8.52	23.24	35.61	1.00
**Aglaonema plant 3**	**LMS400**	0.87	3.75	8.37	1.00
	**LiDARPheno**	5.47	11.16	18.22	1.00
**Canola plant 1**	**LMS400**	0.14	23.56	39.77	0.8
	**LiDARPheno**	7.99	28.97	50.70	0.6
**Canola plant 2**	**LMS400**	0.32	11.36	30.22	0.9
	**LiDARPheno**	0.51	14.73	39.40	0.7
**Canola plant 3**	**LMS400**	3.88	23.57	57.49	1.00
	**LiDARPheno**	3.89	20.83	59.57	1.00

It is evident from the Table 1 that leaf length estimations using the developed LiDARPheno system data are reasonably comparable to the one acquired using the LMS400 commercial LiDAR. However, the LiDARPheno acquired point cloud is not as dense and due to the density of the point cloud, some of the leaves are not detected or filtered out in the filtering algorithm. These leaves in an arbitrary wild plant are leaf numbers 9, 10, and 12. If looked carefully in Figure 8A, those leaves are occluded by another leave, or they are inclined, i.e., due to the inclination angle, LiDARPheno was not able to capture enough number of points to be considered by algorithms to be an object.

The mean (average) error rate for the LiDARPheno and LMS400 are quite similar, LMS400’s mean error rate for estimation of the leaf length is 8.33% while that of the LiDARPheno is 11.86%. The maximum error rate for the leaf length estimation using the LiDARPheno data was 29.7% while for the LMS400 it was 27.83%. The minimum error rate of estimating the leaf length is about 1.03% for the LMS400 data, and 2.24% for the LiDARPheno acquired data. Similarly, the error rates are calculated for all the plants in both the experiments.

**FIGURE 9:**
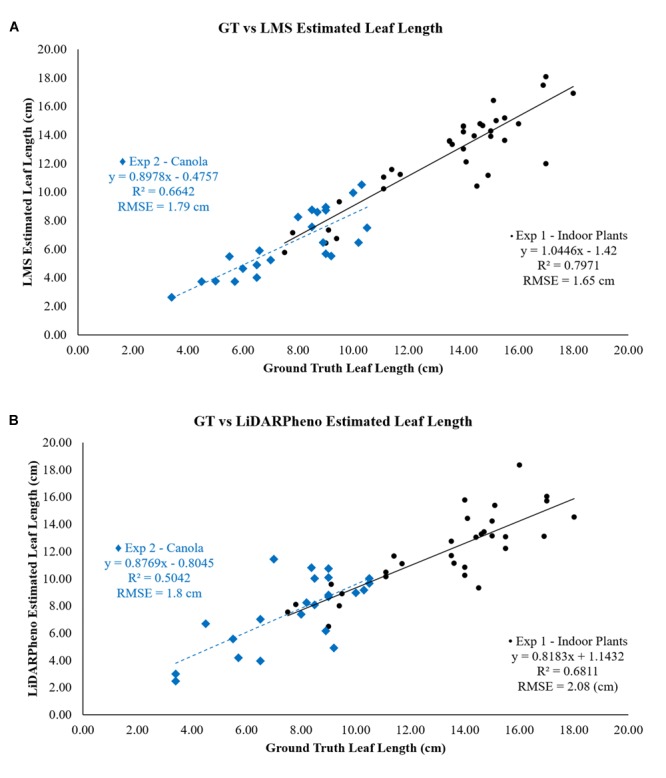
Relation of **(A)** LMS400- and **(B)** LiDARPheno-derived Leaf lengths with Ground truth data.

Figure 9 shows the relation between the estimation using LMS400 data and the ground truth leaf length. The data acquired with LMS400 for experiment 1 on indoor plants show a good relationship between the two with *r*^2^ = 0.7971 and RMSE of 1.65 cm. Experiment 2 on the canola plants, however, has an *r*^2^ = 0.6642 and RMSE = 1.79 cm.

On the other hand, the estimate for the leaf length from the LiDARPheno data indicates RMSE of 2.08 and 1.8 cm for experiments 1 and 2, respectively. Moreover, the coefficient of determination (*r*^2^) for experiments 1 and experiment 2 is *r*^2^ = 0.6811 and *r*^2^ = 0.5042, respectively.

### Leaf Width Estimation Results

**FIGURE 10:**
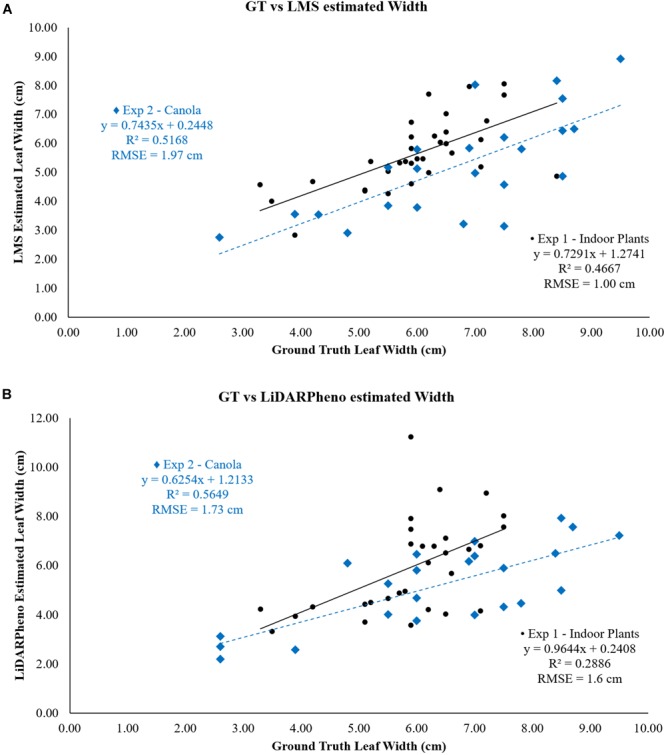
Relation of **(A)** LMS400- and **(B)** LiDARPheno-derived Leaf widths with Ground truth data.

The relationship between the ground truth data and the estimated leaf width with LMS400 and LiDARPheno data is shown in Figure 10A,B. The estimation of the leaf width using LMS400 data has RMSE of 1 cm in experiment 1 and RMSE = 1.97 cm for experiment 2. Coefficient of determination *r*^2^ = 0.47 and *r*^2^ = 0.5168 was achieved for experiments 1 and 2, respectively.

On the other hand, estimation of the width using LiDARPheno data have RMSE = 1.6 cm for plants in experiment 1 and RMSE = 1.73 cm for plants in experiment 2. The correlation coefficients *r*^2^ are 0.29 and 0.56 for experiments 1 and 2, respectively.

### Leaf Area Estimation Results

**FIGURE 11:**
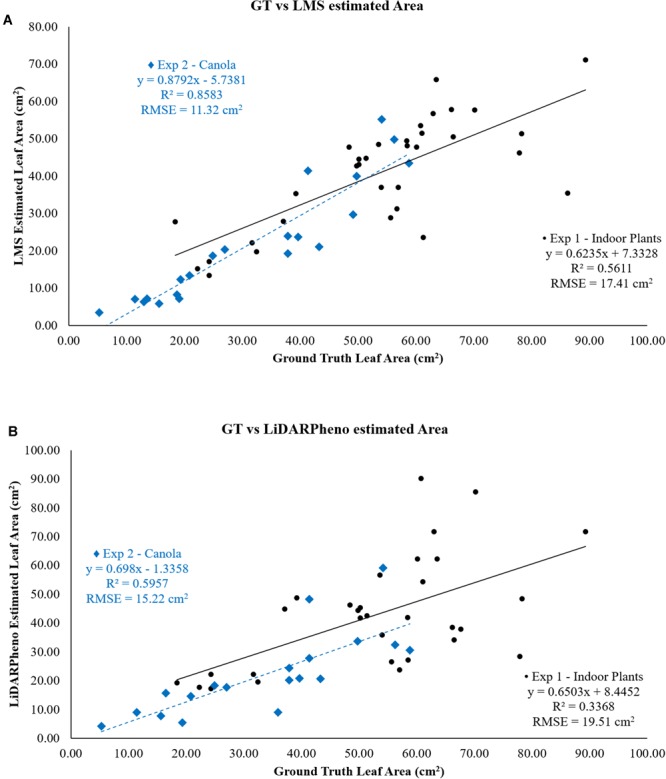
Relation of **(A)** LMS400- and **(B)** LiDARPheno-derived Leaf area with Ground truth data.

The relation between LMS400 estimated area and ground truth leaf area as shown in the plot of Figure 11A. For the experiment 1, *r*^2^ is 0.5611 and RMSE of 17.41 cm^2^. The experiment 2 on canola plants shows a better correlation with the ground truth leaf area with *r*^2^ = 0.8583 and RMSE of 11.32 cm^2^. This suggests the LMS400 is able to correctly estimate the leaf area for the values below 60 cm^2^ and more than that it fails to estimate the leaf area correctly. However, the quality of data acquisition is also dependent on the plant material. For example, the better reflection is necessary for any LiDAR sensor to correctly estimate the distance to that plant, which results in the point cloud.

The leaf area computed using the LiDARPheno point cloud data, and ground truth are related using the scatter plot and linear regression in Figure 11B. Experiment 1 on the various plants of three different species has an RMSE of 19.51 cm^2^ when compared to the ground truth leaf area while *r*^2^ = 0.3368. For experiment 2 on the canola plants, the leaf area estimation results using the LiDARPheno data are compared to the ground truth, and the RMSE of 15.22 cm^2^ is achieved. Moreover, the *r*^2^ of 0.5957 shows good relation to ground truth data.

### Comparing LiDARPheno and LMS400 Derived Results

Table 2 shows the comparison of the developed LiDARPheno system with the LMS400-based 3D scanning system. The first and most important parameter in comparison is the cost of the system. The cost to build LiDARPheno is almost 96% less than the LMS400 device itself. Moreover, the LMS400-based system requires external setup to acquire the 3D point cloud data, while LiDARPheno is an independent system. The LiDARPheno system is much more lightweight than the LMS400-based setup for data acquisition. The power requirement for LMS400 is 25 Watts compared to about 3 watts for LiDARPheno, and hence the small rechargeable LiPo battery can power the LiDARPheno system. LiDARPheno, due to its low-resolution, acquires relatively fewer points and hence, has small file-size. Consequently, the post-processing of the LiDARPheno data is faster compared to the LMS400-based system. Authors were unable to find any related research material to compare the obtained results. Hence, the comparison between commercial and developed system were presented.

**Table 2:** Comparison of the LMS400-based system with LiDARPheno.

	LMS400–based system	LiDARPheno
**Material cost**	∼ $10,000	**∼ $400**
**Scan ready?**	No (requires external setup to hold the LiDAR and move it along scan direction)	**Yes** (the LiDARPheno is designed to work independently of any external requirements)
**Setup**	Bulky (∼1.5 kg for LMS400)	**Light-weight** (Less than 500 g)
**Battery powered?**	Could be (requires large battery)	**Yes** (can run for up to 5 h on 7.4 V 2 Ah LiPo battery)
**Scan time (for 1 m × 1 m)**	**∼5 s**	∼16 min
**File Size (for 1 m × 1 m^2^)**	∼10 Mbytes	**∼300 Kbytes**
**Point cloud density** **(for 1 m × 1 m)**	**∼2.4 Million points**	∼40,000 points
**Post-processing computational complexity**	Highly complex (due to dense point cloud)	**Relatively simple**
**Do-it-yourself (DIY)?**	No (sound technical knowledge is required to acquire data)	**Yes** (The system can be bilt by anyone with little technical knowledge)
**Other external equipment?**	Yes (an external computer is required to acquire the data)	**No** (the system itself has a mini-computer in the design)

*Bold values represent superior feature of either system.*

The LiDARPheno is designed so that anyone with a little or no technical knowledge can build it using the widely available off-the-shelf components used in the system. On the other hand, even though LiDARPheno has many benefits, the LiDARPheno is a slow system due to use of two servo motors and the LiDAR sensor used itself. Hence, the LiDARPheno may take up to 16 min for the scan of 1 m^2^ area, while the LMS400-based system can scan the same area in about 5 s. Moreover, the density of the acquired point cloud using LiDARPheno does not permit the analysis of the smaller areas of the object. Also, availability of the reflectance information from the LMS400 device can be used in many cases which are not provided by the LiDARPheno.

**FIGURE 12:**
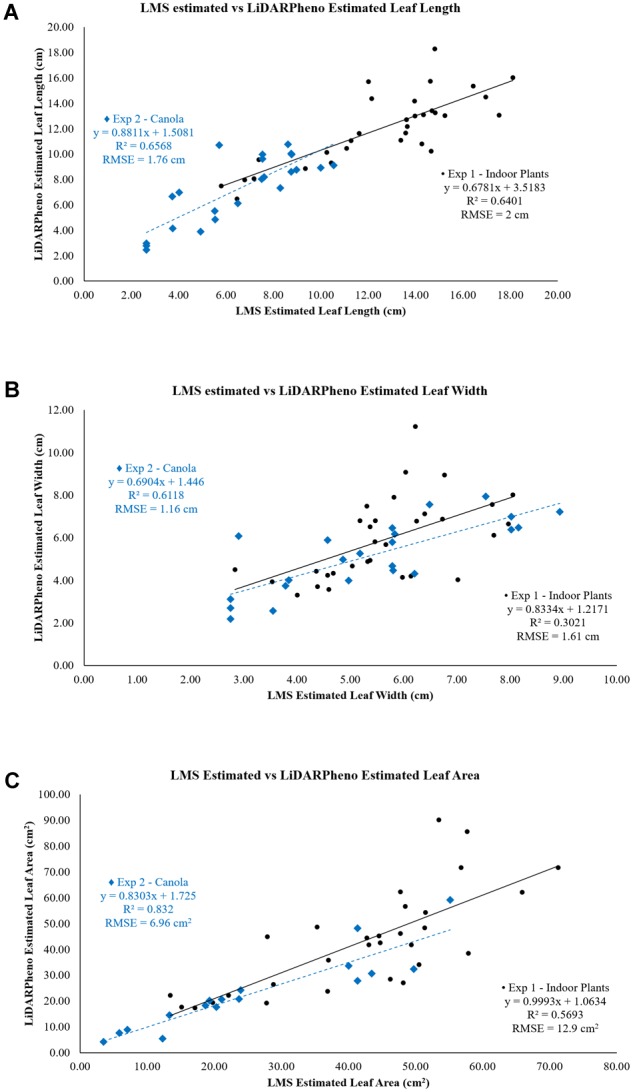
Relation of LMS400- and LiDARPheno-derived leaf length **(A)**, leaf width **(B)**, and leaf area **(C)**.

The comparisons of the results derived with two different systems, LiDARPheno and LMS400, are presented using the correlation plots of the trait estimation data Figure 12A shows the comparison of the LMS400 and LiDARPheno derived leaf length; Figure 12B shows the comparison of the leaf width extraction using two different data, and the relation between the leaf areas estimated using the two systems is presented in Figure 12C. The relation can be determined using *r*^2^ and RMSE between two results.

The leaf length estimation using the commercial LiDAR system LMS400 and LiDARPheno data has a good correlation with the *r*^2^ of 0.64 and 0.66 for experiments 1 and 2, respectively. Moreover, the RMSE of 2 cm in experiment 1 and 1.76 cm in experiment 2 was achieved. The leaf length measurements relation between LMS400-derived and LiDARPheno-derived results indicate that there is a reasonable level of agreement between results estimated using two different data obtained with two different LiDAR sensors.

In Figure 12B, the relationships between the leaf width measurements using the LMS400 and LiDARPheno data is compared using the linear regression plot. The RMSE of 1.61 cm for experiment 1 and 1.16 cm for experiment 2 indicates the error of estimation in cm. However, the correlation between the two is *r*^2^ = 0.3021, and *r*^2^ = 0.6118 for experiments 1 and 2 are presented. This indicates the feasibility of the developed LiDARPheno system to compete with the commercial LiDAR system. The agreement in results of experiment 2, where relatively small canola leaves were scanned, is more satisfactory than the leaf length.

The leaf area measurement agreement between the two LiDAR-based systems is shown in Figure 12C. The leaf area measurements with both the systems show a functional relationship between the two LiDAR data. For the experiment on indoor plants, the *r*^2^ = 0.5693 and RMSE of 12.9 cm^2^ are achieved, and experiment on canola plants show *r*^2^ = 0.832 and RMSE = 6.96 cm^2^. The relation of the estimating the leaf area using the 3D point cloud data is entirely satisfactory. The results on a canola show excellent agreement for leaf area extraction using LMS400 and LiDARPheno.

Overall, the LiDARPheno system is an excellent combination of cost-feature trade-off. With just a fraction of the cost for a commercial LiDAR-based scanning system, the LiDARPheno enables to monitor some of the critical characteristics of the plants while losing some details. The combination of multiple LiDARPheno systems might prove beneficial and may surpass the results of the commercial LiDAR-based systems.

## Conclusion

In this work, a new low-cost, accessible LiDAR-based technology is developed. A miniature version of the “LiDARPheno” is designed and developed with low-cost, off-the-shelf components and modules. Moreover, the design included the use of the wireless communication for the actual remote operation of the device with the feasibility of deploying the device in the greenhouse as well as field environment. Use of the existing libraries and APIs provide the feasibility for non-technical users to build and operate a system. The experimental setup consisting the commercial LiDAR was presented, and a low-cost ground-truth leaf area acquisition method was developed. A method of conversion from raw LiDAR data to the Cartesian coordinates to generate a point cloud was discussed. Simple algorithms for cleaning and segmenting the point clouds were developed and presented. The simple operation of the algorithms helps the user in developing the software for analysis. The high correlation between the estimates of leaf traits with commercial LiDAR and the developed LiDARPheno system was achieved. Moreover, the estimation of the leaf traits using the developed methods shows considerable accuracy. Performance analysis for the developed system and methodologies were carried out in this work to provide the utility of the low-cost system in plant phenotyping tasks.

Finally, this work shows the utility of low-cost LiDAR device in the plant phenotyping tasks. The leaf length, width, and area were estimated using the developed methods for the traits characterization. This work also compared the performance of the developed system with commonly used LiDAR sensor for phenotyping. The developed prototype shows the utility and advantages of the low-cost devices in the plant phenotyping research. Devices developed with the aim of the low-cost system can help fill the gap of the plant phenotyping research and provide opportunities for the researchers in the field to explore the possibilities to 3D imaging and may lead to findings that are entirely novel. This work presents a good trade-off between cost and accuracy of results for leaf trait extraction.

Despite having many benefits, the developed system and methodologies have considerable opportunities to explore the possibilities and improving the methods. For instance, a high-speed low-cost LiDAR can be incorporated to improve the scanning speed, 3D point cloud segmentation algorithms can be explored for better leaf characterization, 3D fusion of the color and LiDAR data can be explored to make a better estimation using the color information for segmentation, and an effort can be put on extracting phenotypes other than presented here.

## Author Contributions

KP designed and developed the system and processing algorithms, performed the experiments, analyzed the data, and wrote this manuscript. AD and KW supervised this work and reviewed this manuscript.

## Conflict of Interest Statement

The authors declare that the research was conducted in the absence of any commercial or financial relationships that could be construed as a potential conflict of interest.

## Footnotes

1 http://wiki.ros.org/sick_lms400

2 https://www.python.org/

3 https://www.dropbox.com/

4 https://www.usa.canon.com

5 https://www.mathworks.com

6 https://www.mathworks.com/help/matlab/ref/delaunaytriangulation.html

7 https://en.wikipedia.org/wiki/Triangle
